# A Novel Contrast Agent Based on Magnetic Nanoparticles for Cholesterol Detection as Alzheimer’s Disease Biomarker

**DOI:** 10.1186/s11671-019-2863-8

**Published:** 2019-01-25

**Authors:** Tamara Fernández-Cabada, Milagros Ramos-Gómez

**Affiliations:** 1 Centre for Biomedical Technology (CTB), Universidad Politécnica de Madrid, Campus de Montegancedo, Pozuelo de Alarcón, 28223 Madrid, Spain; 2 CIBER de Bioingeniería, Biomateriales y Nanomedicina (CIBER-BBN), Madrid, Spain

**Keywords:** Cholesterol, Alzheimer’s disease, Magnetic nanoparticles, Early biomarker

## Abstract

**Background:**

Considering the high incidence of Alzheimer’s disease among the world population over the years, and the costs that the disease poses in sanitary and social terms to countries, it is necessary to develop non-invasive diagnostic tests that allow to detect early biomarkers of the disease. Within the early diagnosis methods, the development of contrast agents for magnetic resonance imaging becomes especially useful.

Accumulating evidence suggests that cholesterol may play a role in the pathogenesis of Alzheimer’s disease since abnormal deposits of cholesterol surrounding senile plaques have been described in animal transgenic models and patients with Alzheimer’s disease. In vivo experiments have also shown that diet-induced hypercholesterolemia enhances intraneuronal accumulation of β-amyloid protein accompanied by microgliosis and accelerates β-amyloid deposition in brains.

**Presentation of the Hypothesis:**

In the present study, we propose for the first time the synthesis of a new nanoconjugate composed of magnetic nanoparticles bound to an anti-cholesterol antibody, to detect the abnormal deposits of cholesterol observed in senile plaques in Alzheimer’s disease by magnetic resonance imaging. The nanoplatform could also reveal the decrease of cholesterol observed in neuronal plasmatic membranes associated with this pathology.

**Testing the Hypothesis:**

Experimental design to test the hypothesis will be done first in vitro and then in ex vivo and in vivo studies in a second stage.

**Implications of the Hypothesis:**

The designed nanoplatform could therefore detect cholesterol deposits at the cerebral level. The detection of this biomarker in areas coinciding with senile plaque accumulations could provide early information on the onset and progression of Alzheimer’s disease.

## Background

Several studies have shown that the presence of an appropriate amount of cholesterol (CHO) in the neuronal plasma membrane plays a key role in protecting nerve cells against the toxicity of β-amyloid protein in Alzheimer’s disease (AD) counteracting the excessive production of this protein [1–3]; neurons enriched in CHO are more resistant against oxidative stress and the toxicity of β-amyloid protein [4, 5].

Therefore, it can be assumed that the amount of CHO present in the neuronal plasma membrane, and not only its plasma levels, may play a role in the pathogenesis of neurodegenerative diseases [6]. In fact, experimental data support the idea that an optimal amount of CHO in cell membranes is necessary to create a protective barrier against toxic agents. A reduced amount of cellular CHO in the plasma membrane alters this protective barrier, reducing the protection against toxic agents, including the β-amyloid protein [7]. Interestingly, neurons in the cerebral cortex of transgenic AD mice contain less CHO in the plasma membrane than those from wild-type mice [8].

Mori et al. [9] showed that both in humans and transgenic amyloid precursor protein (APP) mice, CHO is abnormally accumulated in mature amyloid plaques but not in diffuse or immature plaques, suggesting that CHO could play a role in the formation and progression of senile plaques. Other subsequent studies found that CHO and apolipoprotein E were present in the core of the fibrillar plaques, but not in the diffuse plaques at an early stage. In more advanced stages of the disease, a higher number of fibrillar plaques immunopositive for cholesterol oxidase were described [10]. The amount of free CHO per senile plaque, determined by mass spectrometry, was similar to the β-amyloid protein burden [8]. This mutual increase in the concentration of CHO and senile plaques in AD could suggest a new pathogenic mechanism of the disease [11]. Moreover, in the brain tissues of AD patients, lipid deposits co-localizing with fibrillar senile plaques have been described using anti-Stokes Raman scattering and 2-photon fluorescence microscopy in Thioflavin-S stained samples [10]. Two lipid morphologies can be observed: lamellar structures and coalescing macro-aggregates of sub-micron sizes. Since the lipid composition/organization varies throughout the plaques, there is clear evidence of close amyloid-lipid interplay in fibrillar senile plaques, rendering them more dynamic compositions than previously thought [12].

Further, in order to detect biomarkers of AD at early stages of the disease, several studies have proposed the use of functionalized magnetic iron oxide nanoparticles (MNPs) as specific contrast agents for magnetic resonance imaging (MRI) for senile plaques [13–15] and ferritin protein [16] detection. The hypointense effect exhibited by these particles in T2 and T2*-weighted sequences provides greater contrast in MRI images. Therefore, the use of MNPs as contrast agents for MRI is a promising method for the early diagnosis of AD.

The present work presents the hypothesis of the use of a new contrast based on biofunctionalized MNPs, for the detection by MRI of abnormal accumulations of CHO in the senile plaques, which can be used as a potential biomarker of AD.

The present work presents for the first time, up to our knowledge, the design of a new contrast agent based on biofunctionalized MNPs for the detection by MRI of abnormal accumulations of CHO in the senile plaques, which can be used as a potential biomarker of AD.

## The Hypothesis

Due to the high incidence of AD among the world population over the years [17], and the associated costs of pathology in health and social terms for countries [17], it is urgent to develop noninvasive tools that allow the detection of early biomarkers for diagnosis and evolution of the disease.

Within the early diagnosis methods, the development of contrast agents for molecular imaging (MI) becomes especially useful. MI combines conventional imaging technologies with molecular probes, which are designed to detect aspects of biochemistry and cell biology that underlie disease progression and treatment response [18–20].

**Fig. 1:**
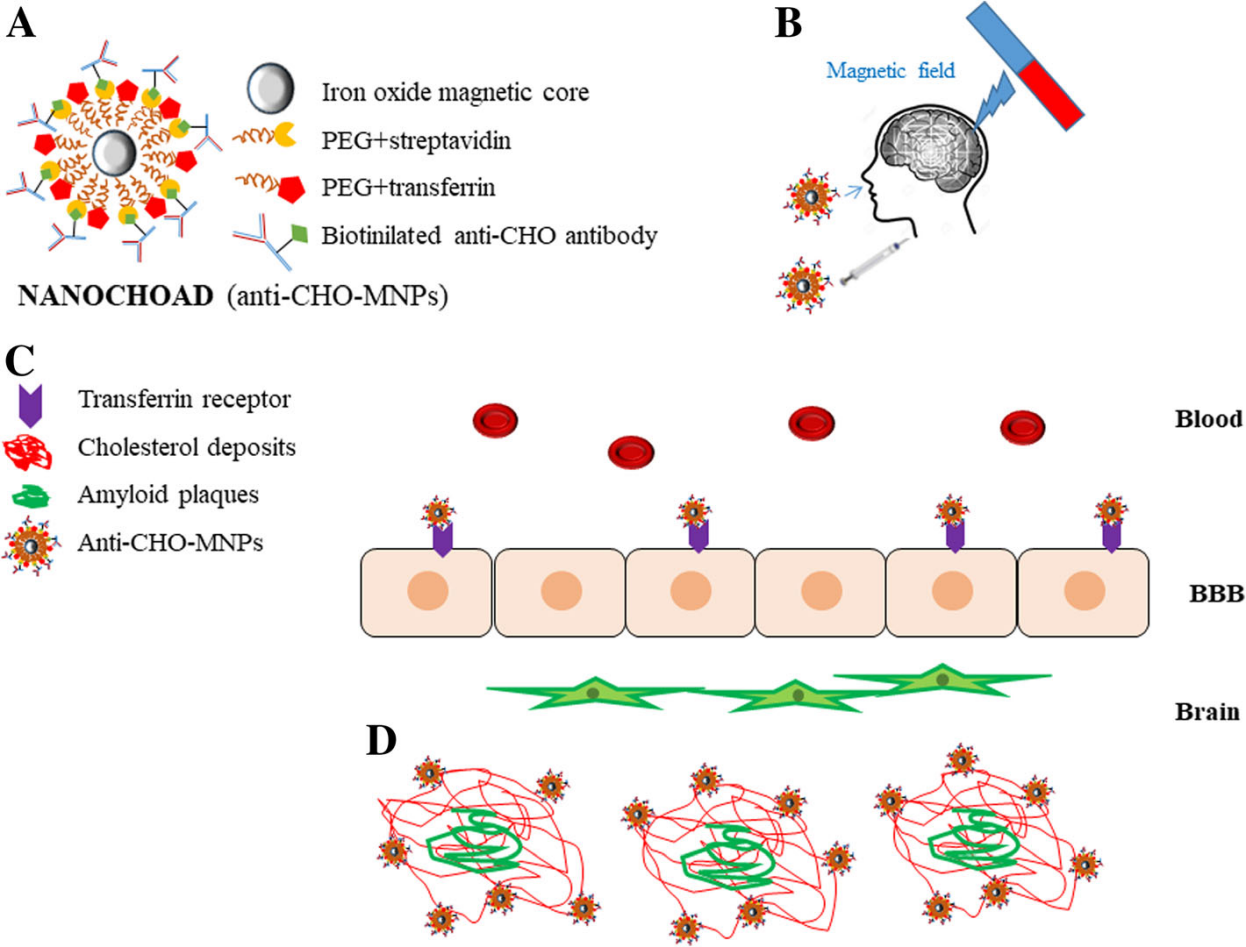
Schematic diagram of the designed nanoplatform and its mechanism of action. **a** the structure of MNP, and functionalization with anti-CHO antibody (NANOCHOAD), **b** strategies for penetration of the nanoplatform through BBB, **c** Crossing mechanism through the BBB of NANOCHOAD. **d** nanoconjugate anti-CHO-MNP targeting CHO deposits on amyloid plaques

We propose the synthesis of a contrast agent based on MNPs coated with polyethylene glycol (PEG) and functionalized with streptavidin (Fig. 1a) to allow the directional linking of a biotinylated antibody that specifically recognizes the CHO present in the senile plaques (NANOCHOAD) (Fig. 1b). The antibody will also recognize the CHO present in the cellular plasma membrane thus detecting the decrease of CHO in neuronal plasma membranes. MNPs will be coated with PEG chains to improve the colloidal stability of the nanoplatform, facilitating its dispersion in the bloodstream and the passage through the blood-brain barrier (BBB) [21].

The BBB represents one of the most exclusive biological barriers encountered in the treatment and diagnosis of neurological diseases, restricting the access of most diagnostic and therapeutic agents to the brain via the systemic route [22, 23]. Therefore, the challenge in diagnosis and treatment of a high number of brain disorders is to overcome the difficulty of delivering therapeutic and contrast agents through the BBB to target concrete regions of the brain. Fortunately, brain capillary endothelial cells show some specific receptor-mediated transport mechanisms. It has been documented that a high number of transferrin receptors are expressed by brain capillary endothelial cells, involved in receptor-mediated transcytosis through the BBB [24].

**Fig. 2:**
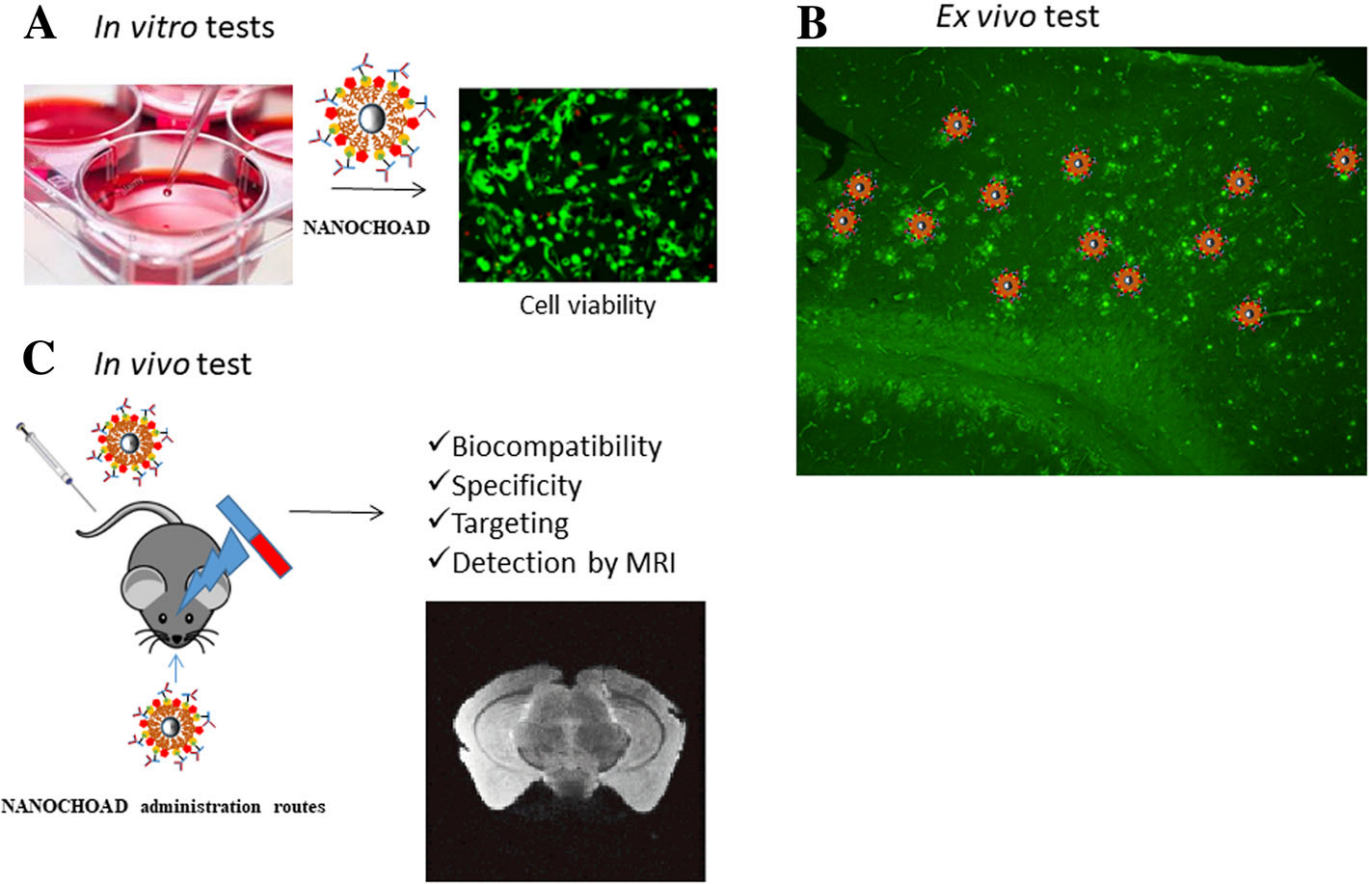
Experimental design to test the hypothesis. First, in vitro (**a**): determination of the biocompatibility of the synthesized nanoconjugate. Ex vivo test (**b**): testing the specificity of anti-CHO-MNPs nanoconjugate by incubation of nanoplatform on fixed brain slices from 5XFAD transgenic mice. In vivo studies (**c**): the nanoplatform will be injected intravenously or by alternative routes as intranasal delivery/external magnetic field application, and its effectiveness (targeting) will be evaluated by MRI

Therefore, to solve the problem of passing the contrast agent through the BBB, we propose three alternative strategies: (i) to conjugate the anti-CHO-MNPs with transferrin [25], which would allow it to go through BBB; (ii) the intranasal administration of the anti-CHO-MNPs conjugate. The intranasal route, which is non-invasive and bypasses the BBB, is an alternative route for delivering nanoconjugates to the brain [26, 27]; and (iii) the application of external magnetic fields to facilitate the nanoconjugate to cross the BBB (Fig. 2c). This novel delivery technique can deliver clinically relevant dosage to the brain (olfactory region, cortex, hippocampus…) through BBB [28–30] (Fig. 1c). Furthermore, the passage of the nanoconjugate through the BBB would also be favored by both the use of PEG-coated MNPs and the deterioration of the BBB due to pathology itself. The nanoconjugate will specifically recognize abnormal deposits of CHO in senile plaques, by the antigen-antibody affinity (Fig. 1c, d). The accumulation of nanoparticles in pathological structures in the brain parenchyma as senile plaques would indicate changes of CHO localization in AD brain parenchyma. Therefore, the presence of anti-CHO-MNPs associated with the senile plaques will show hypointense signals in T2*-weighted MRI, allowing the detection of the CHO associated with other established hallmarks of the AD, such as senile plaques, and thus MR imaging of brain CHO could become a new biomarker of the disease. In addition, changes in MRI due to a decrease in CHO plasma membrane are expected.

The clearance of the contrast agent from the brain parenchyma could be accomplished by the internalization of the MNPs by microglial cells and their subsequent lysosomal processing, as demonstrated in previous studies [16, 31, 32]. MNPs will be eliminated by the common routes used for the metabolism of endogenous iron. Nevertheless, the elimination pathways of the contrast agents, based on their size and surface charge, and their potential toxicity will be determined during the development of the proposed hypothesis, as detailed below.

## Testing the Hypothesis

### Synthesis and Characterization of Magnetic Nanoparticles

The synthesis of iron oxide nanoparticles will be carried out using a controlled co-precipitation method, by mixing ferrous ion (Fe2+) and ferric ion (Fe3+) in alkaline solution following the work from Predescu et al. [33]. MNPs will be coated with a PEG shell following the protocol previously established by Liu et al. [34].

The structure, morphology, and magnetism of the iron nanoparticles coated with PEG will be investigated using X-ray diffraction (XRD), scanning electron microscopy (SEM), transmission electron microscopy (TEM), Fourier-transform infrared spectroscopy (FTIR), and superconducting quantum interference device (SQUID) magnetometry.

After characterization of the synthesized magnetic nanomaterial, it will be functionalized with (1) streptavidin protein by NHS/EDC method. After this, it will be coupled to a biotinylated anti-CHO antibody and (2) transferrin protein. Transferrin conjugation will be carried out by coupling carboxylic group present on the surface of ligand and hydroxyl group present on the PEG coating [35].

### In Vitro Testing

In a first step, the biocompatibility of the nanoplatform will be tested in vitro (Fig. 2a). The nanoconjugate will be added to co-cultures of neurons and astrocytes and to endothelial cell cultures [36] to determine the compatibility of the nanoconjugate with typical brain cells. If the cytocompatibility of the functionalized MNPs is correct, the effectiveness of the system will be tested in an ex vivo model of the disease (Fig. 2b). The selected model is an APP/(presenilin-1) PS1 double transgenic mouse that coexpresses five familial AD mutations (5XFAD) and exhibits an amyloid plaque pathology similar to that found in AD [37].

The accumulation of CHO around the senile plaques in the 5XFAD transgenic model will be evaluated by immunohistochemistry.

If the model is valid, once the accumulation of CHO in the senile plaques is demonstrated, it is proposed to test the specificity of the synthesized nanoconjugate. For it, first, fixed brain slices will be obtained from the 5XFAD transgenic mice, and then, it will be tried on them the union of the nanoconjugate, incubating the anti-CHO-MNPs on the fixed brain sections from 5XFAD mice. The specificity of the nanoconjugate will be determined, by evaluating the colocalization of the anti-CHO-MNPs with the CHO deposits and the senile plaques present in the 5XFAD brain sections, performing the appropriated controls. In case that no evident cholesterol accumulations in 5XFAD were detected, an alternative Swedish mutation on amyloid precursor protein mouse model (APP_sw_), mouse model of AD, will be used, since in this model, an accumulation of CHO clearly associated with amyloid plaques has been described in the hippocampus [9].

### In Vivo Testing

Once the specificity of the conjugate is demonstrated, the biocompatibility analyses of the nanocojugate in vivo will be performed. The nanoplatform would be injected intravenously at different doses (ranging from 25 to 100 mg/kg [38]) (Fig. 2c), and the sub-acute toxicity during the course of the study will be analyzed by observing mortality, evidences of atrophy, congestion, inflammation, or any gross behavioral changes in mice. The weight coefficient of each organ to the body will be calculated. Renal toxicity will be determined by levels of urea nitrogen and creatinine in the blood. The levels of total bilirubin and alkaline phosphatase in blood could be tested as a measure of hepatic and biliar functionality. In addition, the levels of uric acid and hematological studies to assess changes on the levels of red blood cells, white blood cells, and hemoglobin will be determined. Finally, in order to search in more detail for possible toxic effects, histological examination of various tissues (kidney, liver, spleen, brain, or lungs) will be performed [39]. The location of the functionalized MNPs in blood, urine, and different organs would be analyzed at 24 h, 72 h, 1 week, 2 weeks, and 1 month after anti-CHO-MNPs injection.

Once the appropriate concentration of MNPs is determined, the nanoplatform will be injected in control and 5XFAD mice, and its effectiveness will be evaluated by MRI (Fig. 2c). If the antibody from nanoplatform does not recognize the antigen in the in vivo system, then the MNPs could be functionalized with phenyl-diyne cholesterol, a compound that in previous studies has been found to be able to bind to CHO accumulations in vivo [40]. The biocompatibility of this nanoconjugate will be assessed as described above for the MNP-CHO nanoconjugate. If intravenous administration route is not effective crossing the BBB, it is proposed alternative administration routes as intranasal delivery or application of external magnetic fields (Fig. 2c).

## Implications of the Hypothesis

The use of biofunctionalized MNPs to detect AD in vivo by MRI has been widely demonstrated in numerous previous studies, by conjugating the MNPs to different peptides: Aβ 1-40 [41], Aβ1-30 [42], Aβ1-42 [15], and anti- Aβ-1-42 antibodies [43]. After intravenous administration in animal models of AD, both senile plaques and vascular amyloid deposits (congophilic angiopathy) were detected by MRI. However, these nanoconjugates are toxic in themselves since the fragments of the amyloid peptide used are neurotoxic (Aβ1-40, Aβ 1-42). In addition, due to their size, they require the co-administration of compounds that facilitate their passage through the BBB.

NANOCHOAD would work as a contrast agent that would allow simultaneous localization of two AD-specific biomarkers: amyloid plaques and loss of CHO in the white matter of the brain [44], avoiding toxicity. Due to the presence of PEG in its structure, it would also facilitate the passage of the nanoplatform through BBB [43].

As can be seen, most studies of these characteristics are oriented towards the detection of senile plaques, one of the main biomarkers of AD but not the only one. Recently, a paper in which ferritin and therefore iron deposits were detected by nanoconjugates based on MNPs has been published [16]. However, this contrast agent for the detection of ferritin has low sensitivity since it is not detected by MRI and only by quantification in specific locations of the brain. The loss of white matter in the brain is a massive phenomenon [44], not localized; therefore, it is thought that the proposed contrast agent could be more sensitive for the early detection of AD biomarkers. It is necessary to promote the development of new contrast agents that can efficiently detect other biomarkers associated with AD in early stages of the disease.

On the other hand, the composition of the proposed nanoconjugate could solve two of the main obstacles to overcome the efficacy of contrast agents injected intravenously: the colloidal stability in the bloodstream and the ability to successfully cross the BBB to reach the target. The functionalization of the nanoplatform with PEG chains will ensure the colloidal stability of the nanoconjugates in the bloodstream [15, 21]. On the other hand, as a strategy to cross BBB, the conjugation of the MNPs with the peptide transferrin [25] will facilitate the recognition of transferrin by specific receptors located in the BBB allowing the nanoconjugate to cross the BBB and bind to its final target. This fact combined with the reduced size of the nanosystem and the alteration of the BBB in patients with AD would facilitate the passage of the nanoconjugate through the BBB.

Due to the novelty in the design of the described nanoconjugate, it will be necessary to study in depth the biocompatibility and the administered dose of the nanoplatform, especially to determine the routes of elimination of the contrast agent of the organism.

## Abbreviations

AD: Alzheimer’s disease
BBB: Blood-brain barrier
CHO: Cholesterol
MI: Molecular imaging
MNPs: Magnetic iron oxide nanoparticles
MRI: Magnetic resonance imaging
PEG: Polyethylene glycol
